# Experts’ perspectives on human gene editing in Switzerland

**DOI:** 10.1007/s12687-024-00757-0

**Published:** 2024-12-19

**Authors:** Jade Berlincourt, Sumanie Gächter, Effy Vayena, Kelly E Ormond

**Affiliations:** 1 Health Ethics and Policy Lab, Health Sciences and Technologies Department ETH Zurich, Zurich, Switzerland; 2 Health Sciences and Technologies Department, ETH Zurich, Zurich, Switzerland

**Keywords:** Human gene editing, Gene therapy, Expert views, Switzerland, Policy recommendations, Regulatory framework, Ethics

## Abstract

Despite many specialized studies on the views of the public or stakeholders who face inherited conditions that may be treatable by HGE, limited studies have focused on experts’ views towards Human Gene Editing (HGE). Therefore, in this study we conducted exploratory interviews with 14 experts (scientists, clinicians, social scientists, lawyers) in Switzerland to assess their views towards HGE and how they expect the Swiss public to view HGE. We found general acceptance of Somatic Gene Editing (SGE), but opinions towards Germline Gene Editing (GGE) were more divided. Participants emphasized patient autonomy and informed decision-making in pursuing gene editing treatments, and described a need for regulation, as with any other new therapy. Only a few participants (mostly lawyers and ethicists) described the regulations that currently prohibit GGE in Switzerland. Some expressed concern that restrictive regulations would lead to healthcare outsourcing and medical tourism to other nearby countries, as it has in the past with other restricted technologies. The analysis explored the unique Swiss context that is shaped by cultural diversity, conservative attitudes towards new medical technologies, and a democratic system that engages the public in policy and law making. Given that our findings identify areas of difference from that published in other countries, we emphasize the value in conducting similar research across different countries in order to achieve a global sense of attitudes towards HGE, so that regulations can be tailored to the diverse needs of citizens around the world.

**Supplementary information:**

The online version contains supplementary material available at 10.1007/s12687-024-00757-0.

## Introduction

Human Gene Editing (HGE) technology has rapidly advanced beyond Clustered Regularly Interspaced Short Palindromic Repeats (CRISPR) to include prime editing, base editing, and many more techniques still in development. Two main approaches exist: Somatic Gene Editing (SGE), which modifies genes in specific tissues or cells of an individual, and Germline Gene Editing (GGE), which modifies gametes or early-stage embryos, aiming for heritable changes. The use of HGE technologies raises numerous Ethical, Legal, and Social Implications (ELSI) that have been explored in the literature, and which overlap significantly to historical discussions regarding gene therapies and other related treatments. These debates involve various stakeholders, including policy experts, scientists, healthcare providers, bioethicists, patients and families with lived experience of genetic conditions, and the public (for example, Persaud et al. 2018; Hoffman-Andrews et al. 2019; Snure Beckman et al. 2019; Hollister et al. 2019; Delhove et al. 2020; van Dijke et al. 2021; van Baalen et al. 2021; Booth et al. 2021; Elliott et al. 2022; Nicol et al. 2023; Houtman et al. 2023; Sawai et al. 2023). In addition to the many policy statements, guidelines and commentaries that exist on HGE (Nuffield Council on Bioethics 2016; National Academies of Sciences, Engineering, and Medicine 2017, 2023; The Royal Society et al. 2020; European Group on Ethics in Science and New Technologies 2021), there are also a handful of empiric studies examining the experts’ views. These studies have highlighted that ethical assessments revolve, at least to some degree, around potential unforeseen long term consequences and safety issues (for example, off target mutations); the distinction between therapy, prevention, and enhancement; as well as the timing of a gene editing procedure and whether it is heritable; all of which can serve as criteria to determine the ethical acceptability of a particular HGE therapy (Taguchi et al. 2019; Armsby et al. 2019; Waltz et al. 2021; Marchant 2021; Sawai et al. 2023). The potential decrease in population diversity, the question of equitable access to HGE therapies, personal and societal attitudes towards disability, and familial dynamics are also discussed in the literature (Boardman 2020; National Academies of Sciences, Engineering, and Medicine 2023). More philosophical topics of discussion include the concept of humanness and whether altering our DNA changes our fundamental human nature. It is important to evaluate societal impacts and promote open dialogues and collaborative decision-making processes in order to ensure individual rights, societal well-being, and ethical standards as HGE technologies progress (Ormond et al. 2019).

Switzerland, like every country, possesses unique characteristics that may influence citizens’ perspectives about HGE. Switzerland is a small country, with approximately 8.8 million inhabitants in 2023 (Switzerland Federal Statistical Office 2023; De Pietro et al. 2015). It has four main linguistic regions [German, French, Italian, Romansh], and the resultant cultural and linguistic diversity influences opinions (De Pietro et al. 2015). The Swiss political system is notable for its direct democracy. Through regular referendums and popular initiatives, citizens vote directly on policies at all political levels (Linder and Mueller 2021). This approach slows policy, but ultimately increases support for new regulations. Finally, Switzerland is wealthy and stable, and major universities, research centers, and hospitals foster the country’s commitment to education and innovative technologies (WIPO 2022), including HGE, making it a medical technology hub.

In Switzerland, the legal framework for human SGE involves a dual approval process. First, Swissmedic, the Swiss authority responsible for authorizing therapeutic products, must approve somatic gene editing trials and, if applicable, grant market release authorization. Secondly, like all therapeutic trials, gene editing trials require local ethics commission approval and gene editing drug production must follow European Good Manufacturing Practice (GMP) standards (Reg 1252/2014). In vivo (SR 810.30, SR 810.305, SR 812.21) and ex vivo gene therapies (SR 810.21 and SR 810.211) are regulated under different laws. On the other hand, the current legal framework for GGE involves a double prohibition. Switzerland ratified the Oviedo Convention from the Council of Europe which forbids “modification in the genome of the offspring”. Additionally, the Swiss Constitution (art 119, adopted in 2015) prohibits research involving HGE, focusing on preserving dignity, privacy, and family (SR 810.11). Consequently, GGE research and clinical applications are restricted in Switzerland.

Investigating expert opinions is one crucial aspect in the design of policies and regulations around new health technologies such as HGE. Experts possess specialized knowledge and insights that can inform policy considerations and decision-making processes. Given the limited studies exploring the perspectives on HGE in Switzerland, this study aims to describe experts’ views towards HGE in Switzerland.

## Materials and methods

This study employed a qualitative study design of semi-structured interviews due to the exploratory nature of the research and the limited existing data on experts’ opinion on HGE in Switzerland. The study was approved by the ETH Zurich Ethics Committee (EK 2022-N-84).

### Subjects

The target population comprised experts in HGE working in Switzerland from various professional backgrounds: scientists, clinicians, bioethicists, lawyers, and representatives from national advisory boards on ethics and/or medical safety. To identify potential participants, SG, a medical student, conducted an extensive internet search across major academic hospitals and universities in Switzerland, using search terms such as “gene therapy”, “gene editing”, “genome”, and “genetics”. Additionally, we also utilized publicly available lists of physicians specializing in medical genetics from the Swiss Society of Medical Genetics (SGSMS list of FMH/FAMH Medical Genetics), a list of the members of the Gene Therapy Working Group (Office fédéral de la santé publique OFSP), and a list of members of the Swiss National Ethics Commission (National Advisory Commission on Biomedical Ethics NEC 1998). Finally, personal networks of the Health Ethics and Policy Lab identified additional potential participants. Invitations to participate in the study (*N* = 185) were sent twice via email in June and July 2022. Recruitment was concluded upon reaching data saturation, defined as the point where no new information arose for several interviews (Constantinou et al. 2017); this occurred after 14 interviews.

### Interview guide

KO, a genetic counselor and empiric bioethics researcher, and EV, a bioethicist, developed an interview guide (Supplemental Materials) based on a review of relevant literature of expert and public views towards HGE. The guide addressed interviewees’ backgrounds, perspectives on SGE and GGE, and the perceived alignment of Swiss professional and public opinions with existing literature.

### Conducting interviews

Semi-structured interviews were conducted using Zoom by two researchers (KO, SG). Interviews were conducted primarily in English and were audio-recorded; one interview was conducted in German by SG (a native speaker). Verbal consent was obtained before the start of each interview.

### Analysis

The audio-recordings were transcribed using Trint.com, an Artificial Intelligence (AI) based transcription program. The German interview was then translated into English using DeepL.com, an AI based translation website. All transcriptions were de-identified and accuracy and content checked by two researchers (KO, SG). The qualitative analysis approach included several steps (Braun and Clarke 2006). Data familiarization was done through a combination of accuracy checking of the transcripts, and development of interview summaries made by SG and JB, a master student in Health Sciences and Technologies. The original codebook was inductively developed by two researchers (KO and SG) and tested on two transcripts. JB and KO then tested and revised the code book on a third transcript. JB coded the 14 interviews using Nvivo software (14.23.0) using the final codebook. To ensure the reliability of the findings, JB and KO jointly coded every third interview (33%) to consensus. The final themes and sub-themes were then identified, discussed and agreed upon by all members of the research team, and representative quotes were selected to support and illustrate each theme. In presenting the quotes, minor edits were made to enhance readability, for example eliminating repetitions observed in oral communication; these were reviewed by the research team to ensure the meaning was not altered.

## Results

Fourteen interviews were conducted, lasting an average of 55 min (34–76 min). Participants included 64% males (*N* = 9), with an average age of 53 (range 34–68 years). Participants included: five scientists, four clinicians, two lawyers, one historian, one nursing researcher, and one bioethicist. Additionally, several subjects are members of various national advisory boards on ethics and/or medical safety. Both Swiss (*N* = 11) and non-Swiss individuals (*N* = 3) were represented.

The qualitative analysis revealed three main themes reflecting the views of our participants towards HGE in Switzerland: (1) SGE is seen as ‘normal therapy,’ while GGE is more divisive (2) different opinions relate to unique cultural aspects in Switzerland, and (3) responses towards previously published ELSI research regarding HGE.

### Theme 1: SGE is seen as ‘normal therapy’, GGE is more divisive

Participants viewed SGE as scientifically well accepted, and mostly felt it was a ‘normal therapy’. Participants named many conditions for which they felt it would be acceptable; this included conditions both with and without existing treatments, and across the lifecycle (both child and adult onset). Some participants also felt it would be acceptable to treat predispositions to adult conditions, such as cancer. Participants mentioned the benefit that SGE would address the root cause of disease.

*European human geneticists are pretty clear that somatic [gene editing] should be fine.* (Scientist & Geneticist)

Participants suggested that SGE should follow the same regulatory procedures as other authorized therapies, including having a favorable risk-benefit ratio and approval process and undergoing the traditional informed consent processes.

“*Like any new therapy [gene editing] has to be subject to preclinical studies*, *clinical trials*,* because it may have some risks.*” (Lawyer & Ethicist).

Finally, participants recognized that SGE would initially have high prices, but they believed that over time this would decrease as it has with other therapies.

“*Most of these procedures are extremely costly*,* but you may think that at some point it* *may become much simpler and cheaper.*” (Scientist, Physician & Committee representative).

On the other hand, opinions regarding GGE varied significantly. Only a few participants (primarily lawyers or ethicists) were familiar with the regulatory framework in Switzerland. They described the “double prohibition” towards GGE in Switzerland and cited the Oviedo convention, which Switzerland has ratified.

*“Unfortunately it’s a prohibition on a constitutional level … to change this prohibition you need a popular vote. You need a double majority by the people and the canton.”*(Lawyer)

Beyond the legal prohibitions, some participants argued that GGE was unnecessary, particularly where existing approaches such as Preimplantation Genetic Diagnosis (PGD) could provide an alternative. Others acknowledged the potential clinical utility of GGE.

*“We can solve most monogenic diseases mainly with PGD. So germline intervention is not required.”* (Lawyer)

*If you have a very severe mutation for a severe disorder that could be interesting.* (Scientist, Physician & Geneticist)

However, if GGE is authorized in the future, participants suggested that experiences related to PGD could serve as a framework for regulating this new technology.

*“This is the same for preimplantation genetic diagnostics as it’s implemented in Switzerland […] and it probably would be a similar procedure. In terms of counseling and individual assessments.”* (Scientist & Ethicist)

### Theme 2: Swiss characteristics that influence views towards HGE

Switzerland’s cultural and linguistic diversity is perceived as playing a significant role in shaping the variation in attitudes towards many things, including HGE.

*“Dxiversity is what is making us basically Swiss. This is part of our DNA.”* (Scientist & Geneticist)

The Swiss public was described as conservative, reluctant, and prudent, with a broader societal value of preserving natural order and skepticism towards altering fundamental aspects of life.

“I*n Switzerland it’s a pretty common feeling that we should not touch … what nature has given us.”* (Lawyer).

Participants also describe a more cautious approach towards discussing and accepting gene-based therapy in the German speaking part of Switzerland compared to other parts of Switzerland. This difference was attributed to historic reasons; at least one respondent alluded to apprehensions about enhancement following World War II and the history of eugenics.

*“My experience is that it’s more difficult in the German part of Switzerland than. In the Latin Switzerland to talk about gene and to make gene based therapy accepted for the general population, for all kinds of good historical reasons.”* (Scientist & Physician A)

Views about parenthood and reproductive issues were also somewhat cautious, and some related this to how people might approach HGE.

*“Switzerland compared to other countries have a rather traditional view [about parenthood] in the sense that mom stays home and daddy gets the money. And it’s changing much slower than in other countries.”* (Scientist & Ethicist).

While describing this cautious approach towards gene editing, participants did not attribute it to religious beliefs for most Swiss people. Some variances exist, for example, religion is seen as having a bigger impact in the Italian speaking part of Switzerland.

*“I wouldn’t say that religion is an issue […] religion is important to some persons, but obviously much less to the [Swiss] general public.”* (Nurse & Scientist).

Finally, Switzerland was described as slower to adopt new technologies than other countries, in part due to the country’s administration structures. This slower decision-making process allows Switzerland to carefully evaluate and consider the potential risks and benefits associated with new technologies. Then, once a decision is made, it is more certain than in other countries.

*“Swiss laws are kind of ... slow to adopt new, very innovative strategies, which maybe could take time. But once they decide that something is okay, we can move forward in a safe manner.”* (Scientist & Physician A).

The involvement of citizens and the aim to achieve a democratic consensus were also seen as defining Switzerland’s adoption of new technologies and the pace of approvals.

*“The entire … voting population in Switzerland can therefore also participate in technology policy decisions”* (Historian, Ethicist & Committee representative).

*“Swiss way, you try to have a compromise, you have to find a solution that would be acceptable for everyone. … Always one or two people who do not agree with this. But at the end, the majority is going to decide.”* (Physician A).

However, participants unanimously expressed concerns that the Swiss population had limited knowledge and misconceptions about HGE, which could impact public policy and regulations if these misconceptions informed voting choices.

*“They don’t understand or some don’t understand this quite well, they fear things that may not be true.”* (Physician B)

*“When we vote, we vote because we are citizens. We don’t vote because we understand the question.”* (Scientist & Geneticist)

Finally, participants expressed significant concern that if Switzerland does not approve gene editing technologies, individuals may seek these therapies elsewhere, in locations that may have less regulation and ethical oversight. Additionally, some participants believe that it’s necessary to consider GGE research, since neighboring countries may engage in it.

*“If suddenly a family comes and tells me, I would like to perform a germline editing so I could stop this burden in my family for the next generation. […] Let’s assume that this technique would be available in Germany and not in Switzerland. And the family tells me, I would like to go to Berlin and perform the technique, it would be hard to say no.*” (Physician A)

### Theme 3: comparing Swiss concerns and values to the literature

At the end of the interview, participants were presented with a summary of social and ethical norms previously described in the literature that may influence views regarding HGE (questions 5–6 in the interview guide). They were asked to provide comments on their personal views and how they believed the Swiss public might view these issues. The summary included the following issues: the influence of views towards human dignity, disability, and parenthood and reproduction; differences between somatic, germline, therapeutic, preventative and enhancement approaches; equity and justice; and issues of the technologic imperative and potential regulatory approaches.

Most participants emphasized the importance of human dignity in policies on HGE.

*“Dignity has played a role in recent years. I really think that’s shaping our political views.”* (Lawyer).

Additionally, some participants acknowledged that disability is defined differently by different people, arguing that treating conditions that individuals consider as normal can be problematic.

*“There is a community with an inherited form of blindness that is very against gene editing because they feel that they don’t really have a disease. The idea of curing this thing that they don’t consider a disease, it stigmatizes them, makes them feel bad because they’re like, there’s nothing wrong with me. On the other hand, I don’t think that just because some people are totally happy with the way they are, does not mean That everyone else should have that same thing. … people will have very different opinions …based on the disease and on their personal experience.”* (Scientist)

The notion of stigma came up in different ways. First, some participants believe that HGE may worsen and perpetuate stigma.

*“It’s a serious concern that pursuing perfection makes defects more severe.”* (Scientist, Physician & Committee representative).

On the other hand, some participants believe that if treatments such as HGE are available, they should be used. Some took this further, stating that since individuals have the freedom to choose whether to accept or reject potential therapies, they should accept the potential stigmatization that goes with each choice.

*“What can I say? If you choose your stigma, you live with it. If you have access to care. Choose the stigma and then mention that they are victims of stigmatization. To me, that is not honest.”* (Physician, Geneticist, Ethicist, Committee representative)

It was acknowledged by many that there are traditional views towards parenthood and reproduction in Switzerland, and as previously mentioned, the notion of not altering nature was supported. While some participants do believe in accepting children ‘as they come,’ others added the recognition that having a sick child or fertility problems may change the way one views things.

*“If you have your first child that dies after two years of suffering, you will probably change your mind about that for your second child. […] it’s easy to say experience children as they come to you as long as they are healthy and perfect.”* (Physician B)

Conversely, others argue that HGE could change the parent-child relationship, and that societal considerations should outweigh individual wishes.

*“Produce such a radical change in the relationship between parents and children just because a very small number of cases could be the only thing, there are other options. …. We have to consider society, not only individual wishes.”* (Lawyer & Ethicist)

Participants agreed that there is a sense of “technological imperative,” whereby society becomes accustomed to a new technology, and gradually slides down a slippery slope of adoption for broader reasons; here they drew comparisons between PGD and what may happen with GGE or enhancement.

*“That technique [PGD] was initially forbidden in most countries, then it was allowed only for very serious diseases. Then it was allowed for more diseases. And in the end, for instance, in Spain, the most recent reform in the law, it is allowed for anything.”* (Lawyer & Ethicist).

*“Once you allow the technique for therapeutic purposes, I think it’s inevitable that will be then used for non-therapeutic purposes … to introduce genetic changes for the non-therapeutic design of children.”* (Lawyer & Ethicist)

However, there was also a sense that some percentage of the public would fear any new technology, and the government’s decisions about them in general.

*“I think this is true for part of the population [technological imperative], but not all. Some of the people will basically understand every step as an extra reason to doubt about, to entertain these conspiracist thoughts.”* (Scientist, Physician & Committee representative).

*“It’s quite striking that people who are afraid of genetic manipulation are the same to be afraid of manipulation of the information. Basically, they are wary of the government. They do not trust democracy.”* (Scientist, Physician & Committee representative).

Finally, participants emphasized the importance of access to these therapies, the impact on healthcare systems, and how guidelines can prevent inequities.

## Discussion

This qualitative analysis describes experts’ opinions about HGE in Switzerland. Participants appear to widely accept SGE as a ‘normal therapy,’ and similar to other treatments they highlight the need for regulation (without naming anything specific), informed consent, and consideration of cost and equity issues. The views of seeing SGE as ‘normal therapy’ and more debate about GGE aligns with the views within existing literature (Waltz et al. 2021). However, these expert opinions on GGE uniquely reflect social context in Switzerland. Although conservative attitudes result in a slower pace of adopting new technologies, this pace might also be the result of commitment to respecting human dignity and nature, as are mentioned in the Swiss Constitution and related gene technology legislation. Despite Switzerland having relatively strict regulations around reproductive technologies, views towards HGE do not appear to be driven by religious views, which have been identified in other regions (Kalidasan and Das 2022; Jibrilla et al. 2024).

Participants mention PGD in relation to GGE in several ways that are informative. First, some participants mention the existence of PGD reduces the necessity for GGE, a notion well described in the literature (Mertes and Pennings 2015; Sullivan-Pyke and Dokras 2018). However, it is also relevant to know that Switzerland implemented PGD in September 2017 (SR 101 (art 119); Martín et al. 2013), and this recent approval might impact participants’ awareness of existing embryo research regulations and their potential influence on GGE in Switzerland. We can also note that neighboring countries adopted PGD and egg donation much earlier than Switzerland. These examples illustrate that Switzerland has been cautious when adopting emerging reproductive medical technologies. Participants noted that Swiss individuals seeking these procedures before they were legal in Switzerland traveled to neighboring countries (Blöchlinger 2022; Barjot 2004; Corveleyn et al. 2008; Bleichenbacher et al. 2010; de Candolle and de Geyter 2011; Mitter and Widmer 2021; Redaktion SÄZ / Rédaction BMS 2022), suggesting a potential for similar ‘outsourcing’ if HGE treatments are not approved.

Understanding context-specific differences in values-based assessments to technologies like HGE is crucial to tailor regulations that align with a country’s cultural, political, and social context. First, Switzerland’s cultural diversity and its conservatism are perceived to contribute to differing attitudes toward HGE, leading to extended decision-making processes. The country’s political system and size contribute to a slower decision-making process, which can then be informed by the outcomes in neighboring countries, ultimately arriving at outcomes widely accepted by the citizens. While Switzerland’s secularity could foster scientific advancements compared to other nations, reproductive regulations took years to be implemented in comparison with other neighboring countries. This conservatism may be attributed to the notion of ‘dignity’, which is enshrined in the Swiss constitution, and the belief that natural order carries moral integrity; these values may be more important to the Swiss than religious beliefs (Toomey 2020). This may also relate to the discussion of disability in the context of HGE, a recurrent topic in the literature, often embodied by the example of gene therapies for blindness, deafness or short stature (Hoffman-Andrews et al. 2019).

This study has several limitations. Firstly, participants may not fully represent the diversity of expert perspectives within Switzerland, particularly since none were from the Romansh-speaking part of Switzerland. Additionally, the participant pool consisted mainly of individuals with clinical or scientific backgrounds, primarily academic, without representation from industry or politicians. Secondly, experts who voluntarily participated in the study may have held stronger or different views towards HGE. To address this self-selection bias, future research should adopt strategies to encourage a broader range of participants, ensuring a balanced and inclusive representation of viewpoints. Lastly, conducting interviews primarily in English, which was generally not the experts’ native language, could have impacted our ability to see nuance in the data.

In conclusion, this qualitative analysis provides valuable insights into the experts’ opinions on HGE in Switzerland and identifies ways that views towards HGE can vary both between and within countries. We argue that conducting similar studies in a wide range of countries is important to understand the contextual factors unique to each country and to tailor laws and regulations accordingly. Moving forward, the project has several next steps. First, we have conducted a large survey of the Swiss public in Fall 2023 and a deliberative democracy event is planned for upcoming years. More broadly, we hope to stimulate public discussions and provide insights to inform the future development of Swiss policies concerning HGE. The ultimate goal is to ensure that the Swiss regulatory framework aligns with Swiss ethical, societal, and scientific views. By considering expert opinions, public perspectives, and engaging in transparent and democratic processes, Switzerland can develop responsible and ethical policies towards HGE.

## Supplementary information

Below is the link to the electronic supplementary material.

ESM 1 (DOCX 2.80 MB)

## Data Availability

Portions of de-identified interview transcripts, the interview guide and the final codebook are available upon reasonable request to the corresponding author.
