# Supplementary material for: Experts’ perspectives on human gene editing in Switzerland
Source: J Community Genet. 2024 Dec 19;16(1):83–90. doi: 10.1007/s12687-024-00757-0 (PMC11950446; doi:10.1007/s12687-024-00757-0)
Supplement: Supplementary file 1 — (DOCX 2.80 MB) [file 12687_2024_757_MOESM1_ESM.docx]

**Supplemental materials**

**Supplemental Methods - Interview guide**

1.  Please tell us a little bit about yourself, your professional background.

1. Tell us also a little bit about your experience around the topic of gene editing or gene therapy in humans.
2. When it comes to **somatic (non-heritable)** gene editing treatments, what are you own personal views about its potential availability

Prompts:

1. In what situations might you support (or not support) somatic gene editing?
2. What sorts of situations/conditions/traits do you think would be acceptable for somatic gene editing, once the treatments become technically feasible?  Who might decide this?  How ?
3. What experiences and attitudes are those views based on?
4. What concerns do you have about the translational process here in Switzerland?
5. What is your impression of how Swiss laws and regulations apply to this topic?  Do you have legal or regulatory concerns in this area?   (Note, it’s fine if they aren’t familiar enough to comment)
6. Do you think your feelings about somatic gene editing are similar to your colleagues?
7. Do you think your feelings about somatic gene editing are similar to the Swiss public? How or how not?  (e.g. have you had patients discuss this with you? chats with friends or neighbors about it??)

4. When it comes to **germline (heritable)** gene editing treatments, what are your own personal views about its potential availability

Prompts:

1. If it were feasible and legal, in what situations might you hypothetically support (or not support) germline gene editing?
2. What sorts of situations/conditions/traits do you think would be acceptable for germline gene editing, if it were technically feasible and legal?  Who might decide this?  How ?
3. What experiences and attitudes are those views based on?
4. What is your impression of how Swiss laws and regulations apply to this topic?  Do you have legal or regulatory concerns in this area?   (Note, it’s fine if they aren’t familiar enough to comment)
5. Do you agree with these current laws, or would you prefer they be reconsidered?
6. If germline gene editing and its related research  were ever to become legal in Switzerland, what do you imagine the key issues would be in trying to translate it to clinical practice?
7. Do you think your feelings about germline gene editing are similar to your colleagues?
8. Do you think your feelings about germline gene editing are similar to the Swiss public? How or how not?  (e.g. have you had patients discuss this with you? chats with friends or neighbors about it??)

Now I want to transition into how people in other countries have viewed gene editing and get your views on how these may or may not be present in Switzerland

5. Some things that people in other countries have listed as playing a role in the above views are things such as the following:

- Views of what defines us as human
- Views of what makes for a ‘good life’
- Social norms and values, for example towards diversity, solidarity, dignity
- Religious traditions
- Views towards parenthood and reproduction (societal expectations)

Are any of these things where you imagine the Swiss views will play a role in attitudes towards gene editing?  If so, how?

6. In the published literature from other countries, some of the major findings about public attitudes towards gene editing are as follows.  As I read each one [usually also pasted into the chat] , I’d love to hear from you if you think that Swiss public will agree or disagree with the statements and if there things about Swiss law or culture that you think will make views of the public different than views in other countries?

1. Somatic treatment for genetic conditions is generally acceptable as long as there is appropriate safety and regulation.  It is seen as important to reduce suffering and improve quality of life, including the psychological or financial impact on the family
2. Using somatic gene editing preventatively has less support.
3. Enhancement is seen as the least acceptable by most people across the world
4. The general public seems to differentiate less between somatic and germline editing than do scientists and bioethicists.
5. National governments and scientists are seen as having important roles in regulation, but neither alone.  There are worries about conflict of interest
6. There is some feeling that over time we get used to new technologies, and other people that worry that this technologic imperative takes us past the ethical worries
7. Some express worry that those remaining with disabilities or who do not undertake gene editing will be stigmatized
8. Some feel that you should ‘experience children as they come to you’.
9. Some people feel strongly that anything related to embryo research or manipulation should be illegal.

Now that we’re starting to wrap up, a few questions that can help inform our future work.

7. We will be conducting a survey of the Swiss public towards human gene editing this summer.  In general the study will assess views towards both somatic and germline gene editing, including things like under which circumstances gene editing might be offered clinically or performed as research, which conditions or traits might be acceptable for screening, and other logistics around offering it.  Is there anything specific that you personally would like to see assessed on this survey?

8. We are planning ahead towards a deliberative democracy event with the Swiss public in 2023 regarding gene editing.  Events like these include public education regarding the topic, prior to deliberation of the attitudes.  From your personal and professional standpoint, what would be some key topics issues to ensure are fairly covered in the educational sessions?

Are you willing to serve as a potential expert at this type of deliberative event?  Can you recommend others who could speak to these topics?  Before we end, is there anything else you want to say or talk about ? Any questions from your end?
